# New Insights into Pathogenesis and Treatment of ANCA-Associated Vasculitis: Autoantibodies and Beyond

**DOI:** 10.3390/antib12010025

**Published:** 2023-03-21

**Authors:** Marino Paroli, Chiara Gioia, Daniele Accapezzato

**Affiliations:** Division of Clinical Immunology, Department of Clinical, Anesthesiologic and Cardiovascular Sciences, Sapienza University of Rome, 00185 Rome, Italy

**Keywords:** ANCA, vasculitis, pathogenesis, B-cells: complement, neutrophils, eosinophils

## Abstract

Anti-neutrophil cytoplasmic antibody (ANCA)-associated vasculitis is a group of rare systemic diseases affecting small-caliber vessels. The damage caused by AAV mainly involves the lung and kidneys. AAV includes three different types: granulomatosis with polyangiitis (GPA), microscopic polyangiitis (MPA), and eosinophilic granulomatosis with polyangiitis (EGPA). Although the different phenotypic forms of AAV share common features, recent studies have shown that there are significant differences in terms of pathogenetic mechanisms involving both the adaptive and innate immune systems. Advances in our understanding of pathogenesis have enabled the development of immuno-targeted therapies. This review illustrates the characteristics of the various forms of AAV and the new therapies available for this disease that can have lethal consequences if left untreated.

## 1. Introduction

Antineutrophil cytoplasmic antibody (ANCA)-associated vasculitis (AAV) is a group of systemic diseases affecting small vessels. AAV causes damage to several organs and tissues, including the upper and lower respiratory tracts, kidneys, nerves, and skin [1]. AAV comprises three main forms: granulomatosis with polyangiitis (GPA), microscopic polyangiitis (MPA), and eosinophilic granulomatosis with polyangiitis (EGPA) [2]. Another type is drug-induced AAV [3]. AAV is characterized by the presence in the serum of ANCA specific for zymoproteins present in the cytoplasm of neutrophils, namely myeloperoxidase (MPO) and proteinase3 (PR3) [4,5]. The frequency of the presence of these antibodies varies widely among different types of AAV [6]. The most serious complications of AAV are diffuse alveolar hemorrhage and renal failure, both of which can be life-threatening [7,8]. The diagnosis of this condition is still based on Watts’ criteria [9] but a new classification has recently been proposed by ACR/EULAR [10,11,12] to highlight differences in pathogenesis and response to treatment between conditions previously considered most similar. The purpose of this review is to discuss new knowledge about the pathogenesis of different forms of AAV and the latest therapeutic approaches for this complex disease.

## 2. Epidemiology

The introduction of the ANCA test about three decades ago made it possible to identify previously undiagnosed cases of AAV. However, epidemiological data on the incidence and prevalence of AAV have always been hampered by the rarity of the disease [13,14]. In recent years, there has been a gradual increase in the number of patients diagnosed as having AAV. Several explanations have been proposed, including climate change, improved classification criteria, more common use of ANCA testing, and increased awareness of the disease. However, the epidemiology of AAV seems to have stabilized in the early 2000s [15]. The incidence of GPA and MPA seems similar, with up to 30 new cases per million population and a prevalence of up to 250 cases per million population according to epidemiological studies [16,17]. EGPA is much rarer, with an incidence of up to four cases per million population and a prevalence of up to 25 cases per million population [18]. Several studies have shown that the peak incidence of AAV occurs in the age group of 60 to 79 years [19,20]. The higher incidence of AAV in elderly individuals may be partly explained by improved testing for ANCAs in serum, which has made it possible to detect previously undiagnosed forms [19,21]. The ratio of male-to-female prevalence was reported as slightly in favor of women [19,22,23,24]. However, this result has not been confirmed by other studies. Therefore, the question of whether there is a sex-dependent susceptibility to the disease has not yet been definitively answered [25].

### Epigenetics and Environmental Factors

Some environmental factors have been reported to be responsible for the induction or course of AAV. These mainly include infectious agents, drugs, and silica dust. Some studies indicate that toxic shock syndrome toxin-1 (TSST-1) produced by some strains of *S. aureus* is a risk factor for GPA recurrence [26]. It has been hypothesized that this bacterium is involved in the pathogenesis of AAV through a mechanism of molecular mimicry [27]. The hypothesis of the possible involvement of *S. aureus* in AAV is also based on the observation of the therapeutic effect of antibiotic treatment in GPA, as reported by some studies [28,29]. The association between silica dust exposure and the development of AAV was first suggested by a comprehensive meta-analysis [30]. In this regard, it was reported that the severity of the disease increased in patients with AAV after the earthquakes in Japan [31,32]. This observation suggested that silica dust in the air due to the destruction and reconstruction of cities may have influenced the course of AAV, especially at the respiratory level. However, no difference was observed in the incidence of AAV before and after the 2011 New Zealand earthquake [33]. Therefore, the role of earthquakes in the onset or flares of AAV has not yet been conclusively demonstrated.

## 3. Classification and Diagnostic Criteria

The first American College of Rheumatology (ACR) criteria for vasculitis including AAV were published in 1990 and included only GPA and EGPA, but not MPA [34]. Subsequently, new definitions from the Chapel Hill Consensus Conference (CHCC) were published in 1994 and then revised in 2012. On this occasion, the classification took into account the new etiopathogenetic knowledge of the different types of AAV. A tree hierarchy was developed, emphasizing that some conditions cannot be classified simply by vessel size, but require the presence of surrogate markers of disease. AAV was recognized as a specific type of small vessel vasculitis in which the surrogate marker is the presence of serum ANCA [2]. This classification revision was preceded by a stepwise diagnostic algorithm formulated by experts and widely used in clinical practice [9]. The presence of ANCA can be assessed by several tests, the first of which to be proposed was the indirect immunofluorescence test on ethanol-fixed human neutrophils [35]. Alternative and more practical methods are enzyme immunoassay and chemiluminescence. In detail, the c-ANCA describes an indirect immunofluorescence pattern that consists of diffuse granular cytoplasmic staining, characteristic of PR3-ANCA, while the p-ANCA pattern consists of perinuclear staining, typical of MPO-ANCA [36,37]. The pattern most commonly associated with GPA although not exclusively, is c-ANCA [38]. Patients with MPA often show a p-ANCA pattern, but they can also exhibit PR3-ANCA [39]. In EGPA, <50% of patients have detectable ANCA. If present, these are typically MPO-ANCA [40]. More recently, diagnostic criteria for AAV have been proposed that aim to provide more accurate distinctions of individual forms based on their clinical features [10,11,12]. It has also been suggested by some authors that the classification of AAV types was based on the specificity of circulating ANCA rather than clinical features [41].

## 4. Pathogenesis

### 4.1. The Role of Genetic

Through genome-wide association studies (GWAS), several genes have been identified that may be involved in AAV susceptibility. In particular, the major histocompatibility complex class II genes appear to play a major role [42,43,44]. In some studies, GPA with PR3-ANCA is significantly associated with *HLA-DP* genes, while the presence of MPO-ANCA is associated with *HLA-DQ* genes [42,43]. It has also been reported that the HLA-DPB1*04 allele is associated with the risk of developing GPA in North America, while the HLA-DRB1*09 allele is associated with GPA with MPO-ANCA in the Japanese population. These differences reflect the predominance of GPA with PR3-ANCA in European white populations, while GPA with MPO-ANCA is more common in Asian populations [45]. Several associations of AAV with non-MHC genes have also been described. These include *PTPN22* [46], *SERPIN1*, *PRTN3*, and *SEMA6A* genes [42,43,44]. It has been reported that the frequency of a single nucleotide polymorphism (SNP) in the *PTPN22* gene is higher in patients with AAV than in healthy subjects. It has also been shown that this mutated variant is associated with increased production of interleukin (IL)-10 characterized by anti-inflammatory activity. This would result in decreased disease activity in patients with AAV bearing this mutation [46]. An SNP near the *SERPIN1* gene is associated with PR3-ANCA-associated GPA resistance [47]. An SNP in the *PRTN3* gene is associated with resistance to developing PR3-ANCA AAV, while an SNP in the *SEMA6A* gene is associated with resistance to developing GPA. [43,44]. Future studies are needed to better clarify how mutations in these genes are involved in the pathogenesis of AAV. Epigenetic modifications of histones and DNA have been implicated in the regulation of the expression of genes encoding for MPO and PR3 [48,49]. Promoter methylation of *MPO* and *PRTN3* genes was found to be negatively correlated with their transcription [49] and is inversely related to disease activity [49].

### 4.2. The Role of ANCA and Neutrophils

Several studies conducted initially in animal models have shown that ANCA play an important role in the pathogenesis of AAV. For example, injection of MPO-ANCAs into wild-type mice can induce necrotizing and crescentic glomerulonephritis (NCGN) [50]. In other experimental models, the presence of MPO-ANCA has been shown to cause pulmonary hemorrhage [51]. These animal models indicate the pathogenicity of ANCAs and not only their utility as biomarkers of disease. To understand the pathogenic mechanism of ANCAs, their interaction with neutrophils is crucial. Neutrophils play a central role in mediating and amplifying tissue damage. In genetically predisposed individuals and with the contribution of environmental factors, proinflammatory cytokines induce neutrophils to express MPO and PR3 antigens on their cell surface making them visible to autoreactive cells of the adaptive immune system [52]. These antigens can then become the target of ANCA. Such antibodies further activate circulating neutrophils that transmigrate through the endothelium and accumulate at the level of the vascular wall. Here, they can release superoxide radicals and oxygen enzymes, molecules that are extremely damaging to the vessels and can cause their necrosis [53]. Damaged vascular endothelium allows plasma to reach perivascular tissue triggering the coagulator cascade and inducing thrombosis of small vessels [54,55]. Neutrophil activation activates by chemotaxis the arrival of monocytes into the tissue, which in turn induces the release of cytokines, other proinflammatory mediators, reactive oxygen species, and lytic enzymes, further amplifying the inflammatory reaction and tissue damage [56]. A key contribution to tissue damage is also made by the formation and release of neutrophil extracellular traps (NETs) associated with neutrophil apoptosis (NETosis). NETs are extracellular fibrillar arrays containing DNA that constitute an important defense tool of neutrophils against extracellular pathogens [57]. Their activation is very harmful to small vessels [58] and is involved in complement activation [59] and ANCA production [60].

### 4.3. The Role of B- and T-Cells

**Figure 1:**
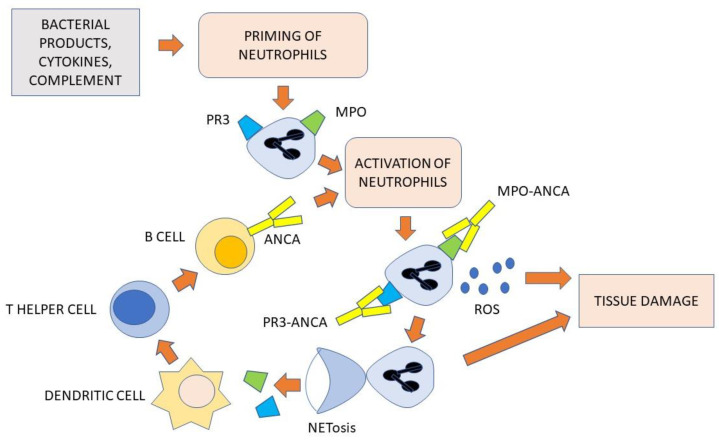
Pathogenesis of AAV. Environmental factors induce neutrophils to express MPO and PR3 on their surface. These cells are then further activated and induce tissue damage through the production of reactive oxygen species (ROS) and the formation of neutrophil extracellular traps (NETs). Exposure of NETs, through a process termed NETosis, is associated with apoptosis of the neutrophils themselves, which release the antigens MPO and PR3 into the extracellular space. These proteins are processed by dendritic cells and then presented to T helper cells. The latter help the B cells to produce ANCA, which further activate the neutrophils thus maintaining the inflammatory process.

MPO and PR3 antigens are discharged in the extracellular environment by neutrophils dying of apoptosis after NETs release. These proteins are phagocytosed by antigen-presenting cells for induction of MPO- and PR3-specific T lymphocytes which are then expanded and activated in the peripheral blood [61]. Abnormal immune responses are thus induced that promote the chronicity of the inflammatory process through the release of cytokines, enzymes, and reactive oxygen species [62]. The T-cell response is mediated mainly by T helper (Th)1 and Th17 cells with pro-inflammatory activity. This process is accompanied by a decrease in circulating regulatory T cells (Tregs) that contribute to the immune reaction against the self-antigens MPO and PR3 [63]. Th17 cells stimulate the recruitment of neutrophils to inflamed sites with the amplification of tissue damage [64]. Conversely, neutrophils provide to induce the enrolment of effector T-cell types and amplification of T-cell memory [65]. Some of these effector T-cells also contribute to natural killer (NK) cell proliferation [66]. Th2 cells provide to help B-cells to produce ANCA [67,68]. Several other factors have been found to contribute to the development of AAV, including defective apoptosis or failure to eliminate apoptotic cells. These may expose self-antigens normally invisible to the immune system [69]. It has also been observed that nasal colonization with S. aureus is often present in GPA, especially in relapsing patients. It has been suggested that this pathogen might contribute to an inflammatory microenvironment necessary for the activation of autoreactive T cells in AAV [70,71]. The direct pathogenicity of ANCA is supported by both experimental and clinical observations [72,73]. In animal models, ANCA have been shown to interact with neutrophils by inducing their degranulation and production of oxygen radicals [74,75]. ANCA can also induce the adhesion properties of neutrophils to the endothelial cells [76,77], inducing vessel wall inflammation of different target organs [50,78,79,80]. The pathogenic role of ANCAs is also supported by clinical data. For example, in drug-associated AAV, remission induced by drug withdrawal is directly related to a significant reduction in circulating ANCA titer [81,82,83]. In support of the pathogenetic role of ANCA, a case of neonatal pulmonary hemorrhage secondary to transplacental passage of MPO-ANCA by the mother has been well described [84]. Another similar clinical case in which an infant of a mother with AAV developed pulmonary hemorrhage and renal kidney damage. The MPO-ANCA assay revealed that the antibody titer in serum was the same as that of the mother. This finding is highly suggestive of the passive transfer of ANCAs by the placental route [85]. More evidence of ANCA pathogenicity includes the observation that targeted therapies that reduce autoantibodies depleting B-cells are effective treatments in AAV [86,87,88]. It is noteworthy, however, that in a mouse model of MPA made B-cell deficient, crescentic glomerulonephritis developed equally in the absence of MPO-ANCA. Depletion of CD4+ effector cells attenuated glomerulonephritis, demonstrating a possible ANCA-independent role of T-cells in the immunopathogenesis of AAV [89]. It has also been reported that regulatory B cells (Breg) induce the trans-differentiation of effector T cells into regulatory T cells (Treg), contributing to reduced ANCA production by B cells. A defect in Breg may therefore be an additional factor promoting the production of AAV [90]. Figure 1 illustrates the pathogenesis of AAV, involving ANCA, neutrophils, dendritic cells, and cells of the adaptive immune system.

### 4.4. The Role of Complement

**Figure 2:**
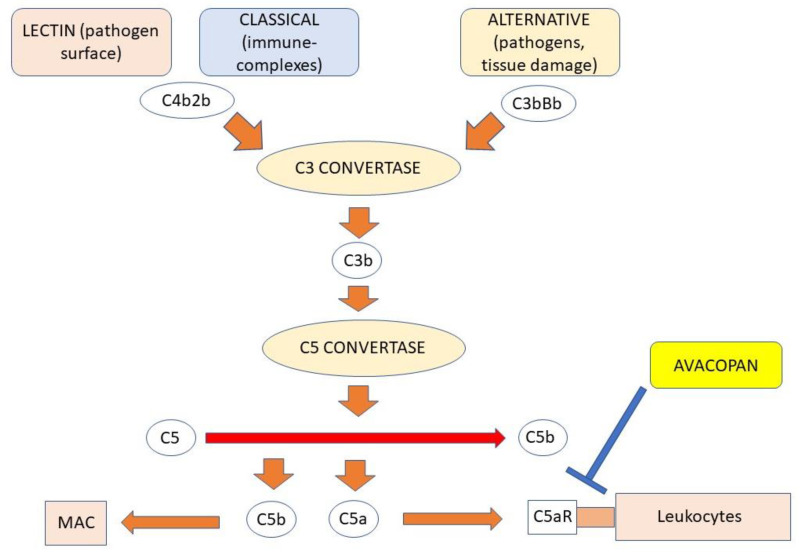
The role of complement. Activation of the alternative complement pathway results by a cascade mechanism in the synthesis of factor C5a, which after recognition of its C5aR receptor on the surface of leukocytes activates them to produce factors that mediate tissue damage. Avacopan, a C5aR agonist, inhibits C5a binding thereby blocking activation of leukocytes including neutrophils.

AAV is considered a pauci-immune disease because immunoglobulin and complement deposits are absent or greatly reduced in patients with AAV [91,92,93]. However, recent experimental and clinical studies suggest that the complement system is actively involved in the pathogenesis of AAV, particularly through the alternative pathway. In this regard, it has been shown that factor B- and C5-deficient mice do not develop the disease after MPO-ANCA administration [94]. On the other hand, the blockade of the C5a receptor (CD88) in mice protects animals from MPO-ANCA-induced vasculitis [95]. Many other studies support the role of C5a factor in AAV pathogenesis [96,97]. Clinical data confirmed that there is an activation of the alternative complement pathway in AAV. Plasma levels of soluble C3a, C5a, C5b-9, and Bb have been described to be higher during active disease than during remission phases [98,99,100,101]. C5a released through the action of C5 convertase can induce mast cell degranulation. This molecule has chemoattractive properties, being able to induce the recruitment of phagocytic cells into tissues and facilitate the migration of antigen-presenting cells into lymph nodes resulting in the activation of the adaptive immune response [102,103,104]. C5a also induces neutrophils to express PR3 on the cell membrane, allowing specific ANCA to bind this protein [105]. In AAV in an active phase, decreased expression of factor H was observed [106]. Factor H not only regulates the alternative complement pathway, but can also bind neutrophils by inhibiting their activation by ANCA. A deficiency of factor H can induce alteration in C3b production, resulting in neutrophil activation with subsequent progression of AAV [107]. Other factors that regulate alternative complement pathways are intercellular adhesion molecule-1 (CD54), decay-accelerating factor (DAF or CD55), and CD59 glycoprotein. The levels of all these proteins can be altered during AAV [108]. Another observation in favor of the role of complement is the evidence that a condition of hypocomplementemia at diagnosis is associated with a worse prognosis and severe renal damage in patients with AAV, due to complement deposition in the small vessels of target organs [109,110]. Figure 2 summarizes the role of complement in the pathogenesis of AAV.

### 4.5. The Role of Eosinophils

EGPA is an AAV characterized mainly by asthma associated with eosinophilia. EGPA can affect several organs, including the skin, lungs, and peripheral nerves [2,111]. EGPA differs mainly from GPA and MPA in the expression of ANCA, which are present in only 30–40% of patients with EGPA [112,113,114]. In addition, some patients with EGPA may not show histological signs of vasculitis and are defined by some authors as having hyper-eosinophilic syndromes (HES) or eosinophilia or suffering from eosinophilic lung disease [115]. EGPA is associated with several immunological dysregulations. CD4+ T-cells, mainly of Th2 phenotype, play a pathogenic role in EGPA. In particular, eosinophils are responsible for most tissue damage [116]. Th2-related cytokines, including IL-5, IL-10, and IL-13, can effectively promote the maturation of eosinophils in the bone marrow and play a key role in their activation peripherally [117]. The success of therapy based on blocking IL-5 by monoclonal antibodies underscores the importance of Th2 cells and eosinophils in disease pathogenesis. In addition, CD4+ T lymphocytes can protect eosinophils from apoptosis, helping them survive longer [118]. Moreover, some studies have revealed that eosinophil proliferation can be triggered by signaling pathways by tyrosine kinases [119]. In addition, endothelial cells can produce eotaxin-3, which can induce the eosinophils to infiltrate tissues and release cytotoxic mediators [120]. Eosinophil cationic protein (ECP) can promote cell death, allowing the presentation of cryptic autoantigens to Th cells, thus perpetuating a vicious cycle [121]. Interferon produced mainly by Th1 cells may also mediate granuloma development, and it has been reported that IL-17 levels, which promote neutrophil recruitment and activation, are significantly increased in EGPA in the active phase [122]. B-cells play also an important role in EGPA immunopathogenesis, as suggested by the therapeutic success achieved with CD20+ B-cell depletion using the monoclonal antibody (mAb) rituximab.

## 5. Clinical Presentation

The clinical features of AAV are heterogeneous and depend largely on the type of AAV considered. These include GPA which primarily affects the upper and lower respiratory tracts, MPA which preferentially affects the kidneys, and EGPA initially characterized by allergy-like symptoms, including asthma, which evolves into definite vasculitis [10,11,12]. The clinical picture and severity are associated with the number of affected vessels, target organs, and disease activity [123]. As this is a systemic disease, patients often present with constitutional symptoms and in particular fever, asthenia, weight loss, and arthralgias [124]. Upper respiratory tract manifestations consist of recurrent nose bleeding, damage to the cartilage of the nasal septum which can collapse, sinusitis, and otitis media [125,126]. Pulmonary manifestations include pulmonary nodules and diffuse alveolar hemorrhage. Alveolar hemorrhage is a particularly severe complication and presents with hemoptysis and dyspnea. An increased incidence of interstitial lung disease has been reported especially in subjects with MPA and MPO-ANCA [127]. The eye can also be affected with several manifestations, among which one of the most frequent is scleritis [128]. In some cases, skin, neurological, or enteric involvement is present [129,130,131]. A more recently recognized co-morbidity is cardiovascular involvement. Cardiovascular disease in AAV is thought to be associated with the acceleration of atherosclerosis. It has been reported that the incidence of cardiovascular events in patients with AAV is three times higher than in the general population, while the risk of cerebrovascular events is increased eightfold compared with healthy controls [132]. An important target organ of AAV is the kidney. The renal disease typically manifests as pauci-immune NCGN. Usually, this condition is characterized by nephritic syndrome with hematuria and proteinuria. Less frequently, renal involvement presents as subacute or chronic nephritis. Renal disease in AAV can progress to end-stage renal failure. Importantly, the frequency of renal involvement differs according to the type of AAV, being higher in MPA than in GPA or EGPA, and is associated with the presence of MPO-ANCA rather than PR3-ANCA [124]. Conversely, involvement of the upper and lower airways is more frequent in patients with GPA than in those with MPA [133]. EGPA presents with a characteristic picture of hypereosinophilia, respiratory allergy, and asthma progressing toward definite vasculitis [134,135].

## 6. Disease Activity

To assess disease activity, the Birmingham Vasculitis Activity Score (BVAS) is commonly used. This score includes 10 categories of symptoms calculated differently depending on whether they are new onset or have worsened for no more than 4 weeks after detection or are present in stable patients. A BVAS score of 0 represents remission, and a BVAS ≥ 1 represents active disease and/or treatment-refractory disease of varying severity. The maximum score is 63 or 33, depending on the patient category considered [136]. The five-factor score (FFS) was validated first for MPA and EGPA and later for GPA. It includes the calculation of serum creatinine, proteinuria, presence of cardiomyopathy, gastrointestinal involvement, and CNS manifestations. It is used to predict the five-year survival rate of AAV patients [137]. The vasculitis damage index (VDI) is a useful clinical tool to distinguish chronic vasculitis-induced damage. To obtain the score, manifestations of the disease must have been present for at least three months. The VDI considers 11 items referring to different organs and systems [138].

## 7. Treatment of AAV

### 7.1. Treatment with Conventional Immunosuppressants

Treatment of AAV has long depended on the use of glucocorticoids and conventional immunosuppressive drugs [88,139]. AAV therapy consists of a first phase for induction of remission and a second phase for maintenance of disease remission. The conventional immunosuppressant commonly used to induce disease remission is cyclophosphamide (CYC) combined or not with steroids. For maintenance therapy, several immunosuppressants, particularly azathioprine, methotrexate, and mycophenolate, are used to spare steroids. CYC is an alkylating agent whose inhibitory effects on B-cells have been recognized, particularly the inhibition of autoantibody production, including autoantibodies [140]. However, serious adverse events secondary to the use of this drug have been reported, including the occurrence of cancer [141,142]. In patients with particularly severe conditions, such as alveolar hemorrhage and rapidly progressive glomerulonephritis, plasmapheresis, which allows rapid and effective removal of ANCAs from serum, may be considered [88]. Regarding maintenance therapy, methotrexate and azathioprine have been found to have similar safety profiles [143]. Mycophenolate was found to be superior to azathioprine in suppressing cytokine production by B-cells in a small cohort of patients [144].

### 7.2. Rituximab

CYC is associated with severe side effects. Therefore, many studies have been devoted to finding safer alternative therapies. Deletion of B lymphocytes to reduce the serum level of ANCA has been considered critical for the treatment of the disease. Research interest has therefore focused on rituximab (RTX), a chimeric murine/human mAb that recognizes and deletes the B lymphocytes [145]. RTX eliminates CD20-expressing B lymphocytes through several mechanisms, including antibody-dependent cellular cytotoxicity (ADCC), complement-dependent cytotoxicity, and induction of apoptosis [146]. The possibility of RTX in inducing remission in AAV has been evaluated in several clinical trials. Two randomized, controlled trials, RAVE and RITUXVAS evaluated the efficacy of RTX for remission induction in GPA and MPA [87,147]. Inclusion criteria for the RAVE study included patients with a diagnosis of GPA or MPA according to the recent AAV definition and positive serum tests for PR3-ANCA or MPO-ANCA with the new onset and relapsing disease but without severe renal failure. The RTX arm was combined with pulse methylprednisolone treatment, and prednisone dosing was reduced to zero within six months. The RITUXVAS study enrolled patients with newly diagnosed vasculitis and more severe renal disease, including patients requiring dialysis. In the RITUXVAS study, the RTX arm received two doses of intravenous CYC and was able to use plasmapheresis. Both studies demonstrated that RTX therapy was non-inferior to CYC therapy for induction of remission, with comparable mortality rates and adverse events. In addition, an analysis of secondary data from the RAVE trial concluded that RTX was superior to CYC in patients with non-severe relapses, who were more likely to be PR3-ANCA positive than MPO-ANCA positive, to have a diagnosis of GPA than MPA, and to have a history of the relapsing disease at baseline [148]. Based on this evidence, current guidelines recommend RTX as the first-line treatment for patients with PR3-ANCA, relapsed disease, refractory disease, and those with contraindications to CYC [88,149]. It should be emphasized, however, that specific measures, such as preventing the reactivation of the hepatitis B virus in patients with occult hepatitis B, controlling antibody production, and preventing the reactivation of latent tuberculosis, are necessary in the case of RTX therapy [150]. In this regard, low levels of IgG class immunoglobulin are observed after RTX therapy in an average of 50–60% of patients [151]. However, in most cases, hypogammaglobulinemia is mild and transient, and IgG levels return to normal within six months of RTX treatment. Only in a small percentage of patients can hypogammaglobulinemia be severe. In that case, intravenous administration of human IgG-class immunoglobulin is required to prevent infectious diseases [152]. Finally, a rare side effect of RTX observed in patients with AAV is sudden and severe neutropenia that occurs within 2–6 months after the last dose of RTX. this event may require the administration of granulocyte growth factors [153]. However, it is necessary to establish the long-term efficacy of RTX as a maintenance therapy. In addition, it should be considered that RTX treatment does not impair the survival of long-lived plasma cells, resulting in the continued production of ANCA, albeit in smaller amounts than in untreated patients [87]. Given the significant toxicity associated with the use of CYC and the relapsing nature of AAV, the use of RTX has nonetheless been approved for the treatment of MPA and GPA as both induction and maintenance therapy [154]. To find safer treatments in AAV therapy and possibly reduce the use of B-cell depleting agents, a study was conducted on the efficacy and safety of plasma exchange in combination with glucocorticoids in patients with severe AAV (PEXIVAS study). However, this treatment approach was not found to reduce the incidence of mortality or end-stage renal disease in treated patients. Therefore, the main conclusion of this study was that the addition of plasma exchange to standard therapy of severe AAV is not indicated [155]. It should be emphasized that the non-approval of RTX for patients with EGPA was because in the studies that led to the drug’s approval such patients were not included [87,147,156]. However, given the role of B cells in the pathogenesis of EGPA, further case series and cohort studies were conducted. The reported results suggest that RTX may also have a role in severe, refractory, or relapsed EGPA, especially if ANCA-positive [157,158,159,160]. The results of two recent systematic reviews have also confirmed the validity of the results obtained from observational studies [161,162]. Response rates similar to those of patients with MPA or GPA were found in patients with EGPA treated with RTX in the European Collaborative Study, a retrospective review of the use of biologics in refractory and/or relapsed EGPA [163]. It is noteworthy that, in most patients with EGPA, RTX has no significant effect on steroid-sparing if asthma is present. Also noteworthy are the results from the REOVAS trial, a randomized, double-blind, controlled trial of RTX in EGPA whose conflicting results still published only as congress abstracts raise doubts about the real efficacy of RTX even in ANCA-positive patients [164]. However, the 2021 ACR/VF guidelines recommend considering RTX for the induction of severe new-onset or relapsing EGPA, particularly in ANCA-positive patients with active glomerulonephritis or at high risk for CYC toxicity if in the absence of cardiac involvement [139]. Conventional immunosuppressants are recommended in the maintenance phase. However, further studies are needed to clarify the role of RTX in EGPA as well as in maintaining disease remission.

### 7.3. C5aR Antagonist Avacopan

Avacopan is an antagonist of C5aR. C5aR is a receptor for C5a that belongs to the G-protein-coupled receptor family. This receptor is expressed on myeloid cells such as granulocytes, macrophages, dendritic cells, mast cells, and various nonmyeloid tissue cells. Activation of this receptor causes inflammation and degranulation of granulocytes, macrophages, and mast cells and vascular permeability as well [165]. The mechanism of action of avacopan is blocking the C11b-induced upregulation of C5a on neutrophils by inhibiting their activation and chemotaxis [95]. In a phase I study, avacopan administered to healthy people produced C5aR inhibition in most subjects at a dose of 30 mg orally twice daily [166]. The phase II, double-blind, placebo-controlled CLEAR trial recruited patients with AAV who were randomized into placebo group with high-dose prednisone, avacopan 30 mg twice daily with low-dose prednisone, or avacopan 30 mg twice daily without prednisone. All patients received standard therapy for induction of remission. The endpoint was a reduction of BVAS ≥ 50% at 12 weeks. This was achieved by 70%, 86%, and 81% of patients in the three groups, respectively [167]. The CLASSIC study, a phase II, randomized, double-blind, placebo-controlled trial, demonstrated the safety and efficacy of avacopan 10 or 30 mg twice daily when added to standard therapy [168]. The ADVOCATE, multicenter, phase III, randomized, double-blind, placebo-controlled trial included patients with AAV randomized to receive avacopan 30 mg twice daily or oral prednisone. These patients also received standard therapy for induction of remission. Maintenance of remission at week 26 indicated that avacopan was non-inferior to prednisone, while sustained remission at week 52 demonstrated the superiority of the avacopan group [169]. Avacopan has thus been approved by the Food and Drug Administration (FDA) and the European Medicines Agency (EMA) for AAV in combination with standard therapy. Eculizumab, a mAb targeting complement protein C5, although not approved for the treatment of AAV, has been effective in refractory cases with an aggressive form of AAV [170,171,172]. Other agents capable of blocking complement are currently being studied. One phase II study evaluated the safety and tolerability of IFX-1, a mAb that binds C5a, in patients with GPA and MPA. The plasma C1 protease inhibitor (C1INH), currently used for the treatment of hereditary angioedema, has also been tested in patients with AAV [173].

### 7.4. The Blockage of Eosinophils in EGPA

**Figure 3:**
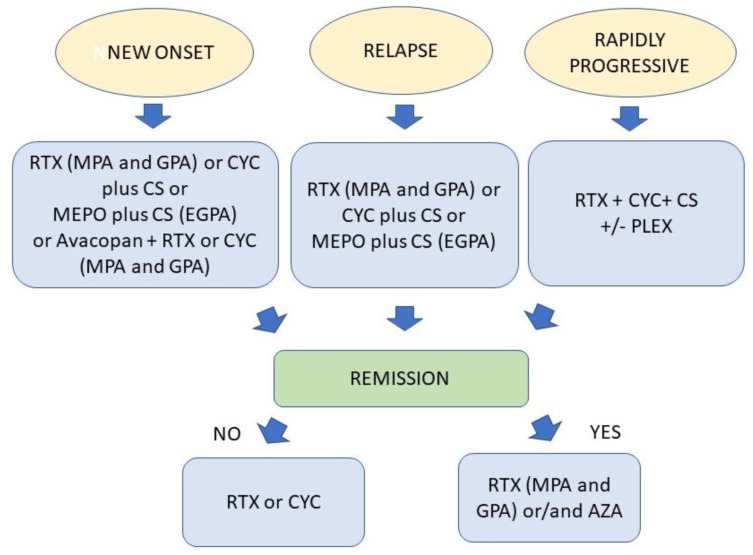
Algorithm for the treatment of AAV. Several strategies are used for induction of remission and maintenance of remission. In addition to conventional drugs such as cyclophosphamide (CYC) corticosteroids (CS) and azathioprine (AZA), new drugs that are able to selectively inhibit immunologic targets such as rituximab (RTX), avacopan, and mepolizumab (MEPO) have been approved by regulatory agencies. PLEX = plasmapheresis. Therapeutic strategies are periodically updated according to new knowledge by EULAR and ACR.

Interleukin-5 (IL-5) is a very important cytokine in the growth, maturation, and differentiation of eosinophils [174]. Mepolizumab is a humanized monoclonal mAb specific for the alpha subunit of IL-5. This antibody blocks the binding of the IL-5 receptor (IL-5R). This mAb widely used in the treatment of asthma was the first drug approved by the FDA for the exclusive treatment of EGPA and no other forms of AAV [175]. The pivotal study that led to the approval of mepolizumab for the treatment of EPGA is the MIRRA study. This double-blind, placebo-controlled study recruited patients with relapsed or refractory EGPA. These were randomized to receive mepolizumab 300 mg subcutaneously every four weeks or placebo in combination with glucocorticoids with or without immunosuppressive therapy. Treated patients achieved the primary endpoint of at least 24 weeks of remission, which was a BVAS of 0. In addition, a 50% lower recurrence rate was observed in the mepolizumab-treated group compared with the placebo group, and a significant reduction in steroid use. However, the MIRRA study included only patients with mild disease. In addition, patients with the presence of ANCA in serum were a minority. This limits the generalizability of the results to ANCA-positive patients. Although a substantial percentage of patients in the treatment group did not achieve remission at 52 weeks, a secondary analysis of the data still showed an absence of flares and confirmed the reduction in glucocorticoid use [175,176]. Several subsequent retrospective studies have confirmed the efficacy of mepolizumab [163,177,178] even at the lower dose of 100 mg per month, such as that used for asthma [163,178]. Two other anti-IL-5 agents currently approved for asthma are being studied in EGPA. Reslizumab, a mAb specific for the IL-5 alpha chain, showed promising results in reducing glucocorticoid use in an open-label pilot study of a small number of patients with EGPA [179]. Benralizumab, a mAb directed against IL-5R, also showed efficacy in treating EGPA in another pilot study. Half of the treated patients stopped taking glucocorticoids at the end of the study [180]. The MANDARA study, which aims to compare benralizumab with mepolizumab in relapsed or refractory EGPA, is still ongoing. Interestingly, this will be the first study to compare head-to-head two biologics for the treatment of EGPA Table 1 shows the main clinical trials conducted on novel AAV therapies. Table 2 shows the drugs approved for the treatment of different phenotypes of AAV and the specific conditions for their use. Figure 3 illustrates the treatment algorithm for the induction of AAV remission and its maintenance.

## 8. Conclusions

Several forms of AAV make such vasculitis a very complex disease. Despite its rarity, it can have lethal effects on the lung and kidneys. Fortunately, several aspects of the immunopathogenesis of AAV have been clarified in recent years. This has led not only to greater knowledge and earlier identification of the disease, but also to the possibility of developing sufficiently effective and safe targeted therapies that can replace traditional immunosuppressants characterized by high toxicity. The main target of therapy remains the inhibition of ANCA production as these autoantibodies play a pathogenic role. RTX was effective in both remission and maintenance of the disease, with far fewer side effects than CYC. However, the discovery of the key role played by alternative complement pathways in MPA and GPA has made C5aR agonist available in the treatment of these types of AAV. Moreover, the anti-IL-5 antibody mepolizumab, already approved for asthma, has been the first drug specifically approved for the treatment of EGPA due to its inhibitory effect on various functions of eosinophils. Future studies will further elucidate the pathogenetic basis of AAV, allowing the design of innovative drugs for an increasingly efficacious and safe therapy.

## Figures and Tables

**Table 1:** Trials on currently approved new drugs for AAV.

Target	Drug	Trial	Primary Endpoint Results
B cells	RTX	RAVE	Non inferiority to oral CYC for remission induction, superior for relapsing or PR3-ANCA patients
B cells	RTX	RITUXVAS	Non inferiority to CYC in pulses for remission induction
B cells	RTX	MAINRISTAN	Superiority to AZA for maintenance of remission
B cells	RTX	MAINRITSAN 2	No difference between standard and customized infusion based on B-cell count for relapse rate
B cells	RTX	REOVAS	Non inferiority to conventional therapy for remission (CYC/CS) in EGPA
C5aR	AVACOPAN	CLASSIC	Safe and effective at day 85
C5aR	AVACOPAN	ADVOCATE	Non inferiority to CS for remission induction
C5aR	AVACOPAN	CLEAR	Non inferiority to CS for remission induction
IL-5	MEPO	MIRRA	Non inferiority to placebo for relapsing or refractory EPGA

RTX = Rituximab; MEPO = Mepolizumab; CYC = Cyclophosphamide; AZA = Azathioprine; CS = Corticosteroids.

**Table 2:** FDA approved indications for new AAV drugs.

Drug	AAV	Specifications
Rituximab	GPA and MPA	In adult and pediatric patients 2 years of age and older in combination with glucocorticoids.
Avacopan	GPA and MPA	As an adjunctive treatment of adult patients with severe active GPA and MPA in combination with standard therapy.
Mepolizumab	EGPA	Adult patients with EGPA.

GPA = granulomatosis with polyangiitis; MPA = microscopic polyangiitis; EPGA = eosinophilic granulomatosis with polyangiitis.

## References

[1] Jennette J.C., Falk R.J. (2014). Pathogenesis of antineutrophil cytoplasmic autoantibody-mediated disease. Nat. Rev. Rheumatol..

[2] Jennette J.C., Falk R.J., Bacon P.A., Basu N., Cid M.C., Ferrario F., Flores-Suarez L.F., Gross W.L., Guillevin L., Hagen E.C. (2013). 2012 Revised International Chapel Hill Consensus Conference Nomenclature of Vasculitides. Arthritis Rheum..

[3] Radic M., Martinovic Kaliterna D., Radic J. (2012). Drug-induced vasculitis: A clinical and pathological review. Neth. J. Med..

[4] Geetha D., Jefferson J.A. (2020). ANCA-Associated Vasculitis: Core Curriculum 2020. Am. J. Kidney Dis..

[5] Kitching A.R., Anders H.J., Basu N., Brouwer E., Gordon J., Jayne D.R., Kullman J., Lyons P.A., Merkel P.A., Savage C.O.S. (2020). ANCA-associated vasculitis. Nat. Rev. Dis. Prim..

[6] Guchelaar N.A.D., Waling M.M., Adhin A.A., van Daele P.L.A., Schreurs M.W.J., Rombach S.M. (2021). The value of anti-neutrophil cytoplasmic antibodies (ANCA) testing for the diagnosis of ANCA-associated vasculitis, a systematic review and meta-analysis. Autoimmun. Rev..

[7] Falk R.J., Hogan S., Carey T.S., Jennette J.C. (1990). Clinical course of anti-neutrophil cytoplasmic autoantibody-associated glomerulonephritis and systemic vasculitis. The Glomerular Disease Collaborative Network. Ann. Intern. Med..

[8] Cartin-Ceba R., Diaz-Caballero L., Al-Qadi M.O., Tryfon S., Fervenza F.C., Ytterberg S.R., Specks U. (2016). Diffuse Alveolar Hemorrhage Secondary to Antineutrophil Cytoplasmic Antibody-Associated Vasculitis: Predictors of Respiratory Failure and Clinical Outcomes. Arthritis Rheumatol..

[9] Watts R., Lane S., Hanslik T., Hauser T., Hellmich B., Koldingsnes W., Mahr A., Segelmark M., Cohen-Tervaert J.W., Scott D. (2007). Development and validation of a consensus methodology for the classification of the ANCA-associated vasculitides and polyarteritis nodosa for epidemiological studies. Ann. Rheum. Dis..

[10] Grayson P.C., Ponte C., Suppiah R., Robson J.C., Craven A., Judge A., Khalid S., Hutchings A., Luqmani R.A., Watts R.A. (2022). 2022 American College of Rheumatology/European Alliance of Associations for Rheumatology Classification Criteria for Eosinophilic Granulomatosis with Polyangiitis. Ann. Rheum. Dis..

[11] Robson J.C., Grayson P.C., Ponte C., Suppiah R., Craven A., Judge A., Khalid S., Hutchings A., Watts R.A., Merkel P.A. (2022). 2022 American College of Rheumatology/European Alliance of Associations for Rheumatology Classification Criteria for Granulomatosis with Polyangiitis. Arthritis Rheumatol..

[12] Suppiah R., Robson J.C., Grayson P.C., Ponte C., Craven A., Khalid S., Judge A., Hutchings A., Merkel P.A., Luqmani R.A. (2022). 2022 American College of Rheumatology/European Alliance of Associations for Rheumatology classification criteria for microscopic polyangiitis. Ann. Rheum. Dis..

[13] Li W., Huang H., Cai M., Yuan T., Sheng Y. (2021). Antineutrophil Cytoplasmic Antibody-Associated Vasculitis Update: Genetic Pathogenesis. Front. Immunol..

[14] Nilsen A.T., Karlsen C., Bakland G., Watts R., Luqmani R., Koldingsnes W. (2020). Increasing incidence and prevalence of ANCA-associated vasculitis in Northern Norway. Rheumatology.

[15] Watts R.A., Hatemi G., Burns J.C., Mohammad A.J. (2022). Global epidemiology of vasculitis. Nat. Rev. Rheumatol..

[16] Mohammad A.J., Jacobsson L.T., Westman K.W., Sturfelt G., Segelmark M. (2009). Incidence and survival rates in Wegener’s granulomatosis, microscopic polyangiitis, Churg-Strauss syndrome and polyarteritis nodosa. Rheumatology.

[17] Hellmich B., Lamprecht P., Spearpoint P., Gotte D., Deichmann A., Buchholz I., Schonermark M.P., Rutherford P. (2021). New insights into the epidemiology of ANCA-associated vasculitides in Germany: Results from a claims data study. Rheumatology.

[18] Pagnoux C. (2010). Churg-Strauss syndrome: Evolving concepts. Discov. Med..

[19] Watts R.A., Lane S.E., Bentham G., Scott D.G. (2000). Epidemiology of systemic vasculitis: A ten-year study in the United Kingdom. Arthritis Rheum..

[20] Pearce F.A., Grainge M.J., Lanyon P.C., Watts R.A., Hubbard R.B. (2017). The incidence, prevalence and mortality of granulomatosis with polyangiitis in the UK Clinical Practice Research Datalink. Rheumatology.

[21] Watts R.A., Lane S.E., Scott D.G., Koldingsnes W., Nossent H., Gonzalez-Gay M.A., Garcia-Porrua C., Bentham G.A. (2001). Epidemiology of vasculitis in Europe. Ann. Rheum. Dis..

[22] Pamuk O.N., Donmez S., Calayir G.B., Pamuk G.E. (2016). The epidemiology of antineutrophil cytoplasmic antibody-associated vasculitis in northwestern Turkey. Clin. Rheumatol..

[23] Berti A., Cornec D., Crowson C.S., Specks U., Matteson E.L. (2017). The Epidemiology of Antineutrophil Cytoplasmic Autoantibody-Associated Vasculitis in Olmsted County, Minnesota: A Twenty-Year US Population-Based Study. Arthritis Rheumatol..

[24] Gonzalez-Gay M.A., Garcia-Porrua C., Guerrero J., Rodriguez-Ledo P., Llorca J. (2003). The epidemiology of the primary systemic vasculitides in northwest Spain: Implications of the Chapel Hill Consensus Conference definitions. Arthritis Rheum..

[25] Salvador F. (2020). ANCA associated vasculitis. Eur. J. Intern. Med..

[26] Popa E.R., Stegeman C.A., Abdulahad W.H., van der Meer B., Arends J., Manson W.M., Bos N.A., Kallenberg C.G., Tervaert J.W. (2007). Staphylococcal toxic-shock-syndrome-toxin-1 as a risk factor for disease relapse in Wegener’s granulomatosis. Rheumatology.

[27] Ooi J.D., Jiang J.H., Eggenhuizen P.J., Chua L.L., van Timmeren M., Loh K.L., O’Sullivan K.M., Gan P.Y., Zhong Y., Tsyganov K. (2019). A plasmid-encoded peptide from Staphylococcus aureus induces anti-myeloperoxidase nephritogenic autoimmunity. Nat. Commun..

[28] Stegeman C.A., Tervaert J.W., de Jong P.E., Kallenberg C.G. (1996). Trimethoprim-sulfamethoxazole (co-trimoxazole) for the prevention of relapses of Wegener’s granulomatosis. Dutch Co-Trimoxazole Wegener Study Group. N. Engl. J. Med..

[29] Salmela A., Rasmussen N., Tervaert J.W.C., Jayne D.R.W., Ekstrand A., European Vasculitis Study G. (2017). Chronic nasal Staphylococcus aureus carriage identifies a subset of newly diagnosed granulomatosis with polyangiitis patients with high relapse rate. Rheumatology.

[30] Gomez-Puerta J.A., Gedmintas L., Costenbader K.H. (2013). The association between silica exposure and development of ANCA-associated vasculitis: Systematic review and meta-analysis. Autoimmun. Rev..

[31] Takeuchi Y., Saito A., Ojima Y., Kagaya S., Fukami H., Sato H., Matsuda K., Nagasawa T. (2017). The influence of the Great East Japan earthquake on microscopic polyangiitis: A retrospective observational study. PLoS ONE.

[32] Yashiro M., Muso E., Itoh-Ihara T., Oyama A., Hashimoto K., Kawamura T., Ono T., Sasayama S. (2000). Significantly high regional morbidity of MPO-ANCA-related angitis and/or nephritis with respiratory tract involvement after the 1995 great earthquake in Kobe (Japan). Am. J. Kidney Dis..

[33] Farquhar H.J., McGettigan B., Chapman P.T., O’Donnell J.L., Frampton C., Stamp L.K. (2017). Incidence of anti-neutrophil cytoplasmic antibody-associated vasculitis before and after the February 2011 Christchurch Earthquake. Intern. Med. J..

[34] Hunder G.G., Arend W.P., Bloch D.A., Calabrese L.H., Fauci A.S., Fries J.F., Leavitt R.Y., Lie J.T., Lightfoot R.W., Masi A.T. (1990). The American College of Rheumatology 1990 criteria for the classification of vasculitis. Introduction. Arthritis Rheum..

[35] Rasmussen N., Wiik A. (1989). Indirect immunofluorescence examination for IgG-ANCA in sera submitted for the 1st international workshop on ANCA, 1988. APMIS Suppl..

[36] Schulte-Pelkum J., Radice A., Norman G.L., Lomicronpez Hoyos M., Lakos G., Buchner C., Musset L., Miyara M., Stinton L., Mahler M. (2014). Novel clinical and diagnostic aspects of antineutrophil cytoplasmic antibodies. J. Immunol. Res..

[37] Savige J., Gillis D., Benson E., Davies D., Esnault V., Falk R.J., Hagen E.C., Jayne D., Jennette J.C., Paspaliaris B. (1999). International Consensus Statement on Testing and Reporting of Antineutrophil Cytoplasmic Antibodies (ANCA). Am. J. Clin. Pathol..

[38] Lutalo P.M., D’Cruz D.P. (2014). Diagnosis and classification of granulomatosis with polyangiitis (aka Wegener’s granulomatosis). J. Autoimmun..

[39] Chung S.A., Seo P. (2010). Microscopic polyangiitis. Rheum. Dis. Clin. N. Am..

[40] Gioffredi A., Maritati F., Oliva E., Buzio C. (2014). Eosinophilic granulomatosis with polyangiitis: An overview. Front. Immunol..

[41] Yazici H., Tascilar K., Yazici Y. (2023). 2022 American College of Rheumatology/European Alliance of Associations for Rheumatology classification criteria sets for three types of antineutrophilic cytoplasmic antibody-associated vasculitis. Curr. Opin. Rheumatol..

[42] Lyons P.A., Rayner T.F., Trivedi S., Holle J.U., Watts R.A., Jayne D.R., Baslund B., Brenchley P., Bruchfeld A., Chaudhry A.N. (2012). Genetically distinct subsets within ANCA-associated vasculitis. N. Engl. J. Med..

[43] Xie G., Roshandel D., Sherva R., Monach P.A., Lu E.Y., Kung T., Carrington K., Zhang S.S., Pulit S.L., Ripke S. (2013). Association of granulomatosis with polyangiitis (Wegener’s) with HLA-DPB1*04 and SEMA6A gene variants: Evidence from genome-wide analysis. Arthritis Rheum..

[44] Rahmattulla C., Mooyaart A.L., van Hooven D., Schoones J.W., Bruijn J.A., Dekkers O.M., European Vasculitis Genetics C., Bajema I.M. (2016). Genetic variants in ANCA-associated vasculitis: A meta-analysis. Ann. Rheum. Dis..

[45] Fujimoto S., Watts R.A., Kobayashi S., Suzuki K., Jayne D.R., Scott D.G., Hashimoto H., Nunoi H. (2011). Comparison of the epidemiology of anti-neutrophil cytoplasmic antibody-associated vasculitis between Japan and the U.K. Rheumatology.

[46] Cao Y., Yang J., Colby K., Hogan S.L., Hu Y., Jennette C.E., Berg E.A., Zhang Y., Jennette J.C., Falk R.J. (2012). High basal activity of the PTPN22 gain-of-function variant blunts leukocyte responsiveness negatively affecting IL-10 production in ANCA vasculitis. PLoS ONE.

[47] Relle M., Fohr B., Fasola F., Schwarting A. (2016). Genetics and pathophysiology of granulomatosis with polyangiitis (GPA) and its main autoantigen proteinase 3. Mol. Cell. Probes.

[48] Ciavatta D.J., Yang J., Preston G.A., Badhwar A.K., Xiao H., Hewins P., Nester C.M., Pendergraft W.F., Magnuson T.R., Jennette J.C. (2010). Epigenetic basis for aberrant upregulation of autoantigen genes in humans with ANCA vasculitis. J. Clin. Investig..

[49] Jones B.E., Yang J., Muthigi A., Hogan S.L., Hu Y., Starmer J., Henderson C.D., Poulton C.J., Brant E.J., Pendergraft W.F. (2017). Gene-Specific DNA Methylation Changes Predict Remission in Patients with ANCA-Associated Vasculitis. J. Am. Soc. Nephrol..

[50] Xiao H., Heeringa P., Hu P., Liu Z., Zhao M., Aratani Y., Maeda N., Falk R.J., Jennette J.C. (2002). Antineutrophil cytoplasmic autoantibodies specific for myeloperoxidase cause glomerulonephritis and vasculitis in mice. J. Clin. Investig..

[51] Little M.A., Smyth L., Salama A.D., Mukherjee S., Smith J., Haskard D., Nourshargh S., Cook H.T., Pusey C.D. (2009). Experimental autoimmune vasculitis: An animal model of anti-neutrophil cytoplasmic autoantibody-associated systemic vasculitis. Am. J. Pathol..

[52] Hellmich B., Csernok E., Trabandt A., Gross W.L., Ernst M. (2000). Granulocyte-macrophage colony-stimulating factor (GM-CSF) but not granulocyte colony-stimulating factor (G-CSF) induces plasma membrane expression of proteinase 3 (PR3) on neutrophils in vitro. Clin. Exp. Immunol..

[53] Van Rossum A.P., Limburg P.C., Kallenberg C.G. (2005). Activation, apoptosis, and clearance of neutrophils in Wegener’s granulomatosis. Ann. N. Y. Acad. Sci..

[54] Ma T.T., Huang Y.M., Wang C., Zhao M.H., Chen M. (2014). Coagulation and fibrinolysis index profile in patients with ANCA-associated vasculitis. PLoS ONE.

[55] Claudel S.E., Tucker B.M., Kleven D.T., Pirkle J.L., Murea M. (2020). Narrative Review of Hypercoagulability in Small-Vessel Vasculitis. Kidney Int. Rep..

[56] Kallenberg C.G. (2011). Pathogenesis of ANCA-associated vasculitides. Ann. Rheum. Dis..

[57] Papayannopoulos V. (2018). Neutrophil extracellular traps in immunity and disease. Nat. Rev. Immunol..

[58] Hattanda F., Nakazawa D., Watanabe-Kusunoki K., Kusunoki Y., Shida H., Masuda S., Nishio S., Tomaru U., Atsumi T., Ishizu A. (2019). The presence of anti-neutrophil extracellular trap antibody in patients with microscopic polyangiitis. Rheumatology.

[59] Wang H., Wang C., Zhao M.H., Chen M. (2015). Neutrophil extracellular traps can activate alternative complement pathways. Clin. Exp. Immunol..

[60] Nakazawa D., Masuda S., Tomaru U., Ishizu A. (2019). Pathogenesis and therapeutic interventions for ANCA-associated vasculitis. Nat. Rev. Rheumatol..

[61] Wilde B., Thewissen M., Damoiseaux J., van Paassen P., Witzke O., Tervaert J.W. (2010). T cells in ANCA-associated vasculitis: What can we learn from lesional versus circulating T cells?. Arthritis Res. Ther..

[62] Hutton H.L., Holdsworth S.R., Kitching A.R. (2017). ANCA-Associated Vasculitis: Pathogenesis, Models, and Preclinical Testing. Semin. Nephrol..

[63] Hilhorst M., Shirai T., Berry G., Goronzy J.J., Weyand C.M. (2014). T cell-macrophage interactions and granuloma formation in vasculitis. Front. Immunol..

[64] Dolff S., Witzke O., Wilde B. (2019). Th17 cells in renal inflammation and autoimmunity. Autoimmun. Rev..

[65] Martinez Valenzuela L., Bordignon Draibe J., Fulladosa Oliveras X., Bestard Matamoros O., Cruzado Garrit J.M., Torras Ambros J. (2019). T-lymphocyte in ANCA-associated vasculitis: What do we know? A pathophysiological and therapeutic approach. Clin. Kidney J..

[66] Perez C., Prajapati K., Burke B., Plaza-Rojas L., Zeleznik-Le N.J., Guevara-Patino J.A. (2019). NKG2D signaling certifies effector CD8 T cells for memory formation. J. Immunother. Cancer.

[67] Vaglio A., Buzio C., Zwerina J. (2013). Eosinophilic granulomatosis with polyangiitis (Churg-Strauss): State of the art. Allergy.

[68] Sanders J.S., Stegeman C.A., Kallenberg C.G. (2003). The Th1 and Th2 paradigm in ANCA-associated vasculitis. Kidney Blood Press. Res..

[69] Reumaux D., Duthilleul P., Roos D. (2004). Pathogenesis of diseases associated with antineutrophil cytoplasm autoantibodies. Hum. Immunol..

[70] Popa E.R., Stegeman C.A., Kallenberg C.G., Tervaert J.W. (2002). Staphylococcus aureus and Wegener’s granulomatosis. Arthritis Res. Ther..

[71] Paroli M., Caccavale R., Fiorillo M.T., Spadea L., Gumina S., Candela V., Paroli M.P. (2022). The Double Game Played by Th17 Cells in Infection: Host Defense and Immunopathology. Pathogens.

[72] Falk R.J., Jennette J.C. (2002). ANCA are pathogenic--oh yes they are!. J. Am. Soc. Nephrol..

[73] Shochet L., Holdsworth S., Kitching A.R. (2020). Animal Models of ANCA Associated Vasculitis. Front. Immunol..

[74] Falk R.J., Terrell R.S., Charles L.A., Jennette J.C. (1990). Anti-neutrophil cytoplasmic autoantibodies induce neutrophils to degranulate and produce oxygen radicals in vitro. Proc. Natl. Acad. Sci. USA.

[75] Keogan M.T., Esnault V.L., Green A.J., Lockwood C.M., Brown D.L. (1992). Activation of normal neutrophils by anti-neutrophil cytoplasm antibodies. Clin. Exp. Immunol..

[76] Radford D.J., Savage C.O., Nash G.B. (2000). Treatment of rolling neutrophils with antineutrophil cytoplasmic antibodies causes conversion to firm integrin-mediated adhesion. Arthritis Rheum..

[77] Taekema-Roelvink M.E.J., Kooten C.V., Kooij S.V., Heemskerk E., Daha M.R. (2001). Proteinase 3 enhances endothelial monocyte chemoattractant protein-1 production and induces increased adhesion of neutrophils to endothelial cells by upregulating intercellular cell adhesion molecule-1. J. Am. Soc. Nephrol..

[78] Heeringa P., Brouwer E., Klok P.A., Huitema M.G., van den Born J., Weening J.J., Kallenberg C.G. (1996). Autoantibodies to myeloperoxidase aggravate mild anti-glomerular-basement-membrane-mediated glomerular injury in the rat. Am. J. Pathol..

[79] Kobayashi K., Shibata T., Sugisaki T. (1995). Aggravation of rat nephrotoxic serum nephritis by anti-myeloperoxidase antibodies. Kidney Int..

[80] Brouwer E., Huitema M.G., Klok P.A., de Weerd H., Tervaert J.W., Weening J.J., Kallenberg C.G. (1993). Antimyeloperoxidase-associated proliferative glomerulonephritis: An animal model. J. Exp. Med..

[81] Tomasson G., Grayson P.C., Mahr A.D., Lavalley M., Merkel P.A. (2012). Value of ANCA measurements during remission to predict a relapse of ANCA-associated vasculitis--a meta-analysis. Rheumatology.

[82] Gao Y., Chen M., Ye H., Yu F., Guo X.H., Zhao M.H. (2008). Long-term outcomes of patients with propylthiouracil-induced anti-neutrophil cytoplasmic auto-antibody-associated vasculitis. Rheumatology.

[83] Chen M., Gao Y., Guo X.H., Zhao M.H. (2012). Propylthiouracil-induced antineutrophil cytoplasmic antibody-associated vasculitis. Nat. Rev. Nephrol..

[84] Bansal P.J., Tobin M.C. (2004). Neonatal microscopic polyangiitis secondary to transfer of maternal myeloperoxidase-antineutrophil cytoplasmic antibody resulting in neonatal pulmonary hemorrhage and renal involvement. Ann. Allergy Asthma Immunol..

[85] Schlieben D.J., Korbet S.M., Kimura R.E., Schwartz M.M., Lewis E.J. (2005). Pulmonary-renal syndrome in a newborn with placental transmission of ANCAs. Am. J. Kidney Dis..

[86] Rovin B.H., Adler S.G., Barratt J., Bridoux F., Burdge K.A., Chan T.M., Cook H.T., Fervenza F.C., Gibson K.L., Glassock R.J. (2021). Executive summary of the KDIGO 2021 Guideline for the Management of Glomerular Diseases. Kidney Int..

[87] Stone J.H., Merkel P.A., Spiera R., Seo P., Langford C.A., Hoffman G.S., Kallenberg C.G., St Clair E.W., Turkiewicz A., Tchao N.K. (2010). Rituximab versus cyclophosphamide for ANCA-associated vasculitis. N. Engl. J. Med..

[88] Yates M., Watts R.A., Bajema I.M., Cid M.C., Crestani B., Hauser T., Hellmich B., Holle J.U., Laudien M., Little M.A. (2016). EULAR/ERA-EDTA recommendations for the management of ANCA-associated vasculitis. Ann. Rheum. Dis..

[89] Ruth A.J., Kitching A.R., Kwan R.Y., Odobasic D., Ooi J.D., Timoshanko J.R., Hickey M.J., Holdsworth S.R. (2006). Anti-neutrophil cytoplasmic antibodies and effector CD4+ cells play nonredundant roles in anti-myeloperoxidase crescentic glomerulonephritis. J. Am. Soc. Nephrol..

[90] Jennette J.C., Falk R.J. (2014). B cell-mediated pathogenesis of ANCA-mediated vasculitis. Semin. Immunopathol..

[91] Dudreuilh C., Fakhouri F., Vigneau C., Augusto J.F., Machet M.C., Rabot N., Chapal M., Charpy V., Barbet C., Buchler M. (2020). The Presence of Renal IgG Deposits in Necrotizing Crescentic Glomerulonephritis Associated with ANCA Is Not Related to Worse Renal Clinical Outcomes. Kidney Dis..

[92] Fauci A.S., Wolff S.M. (1994). Wegener’s granulomatosis: Studies in eighteen patients and a review of the literature. 1973. Medicine.

[93] Van Paassen P., Tervaert J.W., Heeringa P. (2007). Mechanisms of vasculitis: How pauci-immune is ANCA-associated renal vasculitis?. Nephron Exp. Nephrol..

[94] Xiao H., Schreiber A., Heeringa P., Falk R.J., Jennette J.C. (2007). Alternative complement pathway in the pathogenesis of disease mediated by anti-neutrophil cytoplasmic autoantibodies. Am. J. Pathol..

[95] Xiao H., Dairaghi D.J., Powers J.P., Ertl L.S., Baumgart T., Wang Y., Seitz L.C., Penfold M.E., Gan L., Hu P. (2014). C5a receptor (CD88) blockade protects against MPO-ANCA GN. J. Am. Soc. Nephrol..

[96] Schreiber A., Xiao H., Jennette J.C., Schneider W., Luft F.C., Kettritz R. (2009). C5a receptor mediates neutrophil activation and ANCA-induced glomerulonephritis. J. Am. Soc. Nephrol..

[97] Hao J., Meng L.Q., Xu P.C., Chen M., Zhao M.H. (2012). p38MAPK, ERK and PI3K signaling pathways are involved in C5a-primed neutrophils for ANCA-mediated activation. PLoS ONE.

[98] Wu E.Y., McInnis E.A., Boyer-Suavet S., Mendoza C.E., Aybar L.T., Kennedy K.B., Poulton C.J., Henderson C.D., Hu Y., Hogan S.L. (2019). Measuring Circulating Complement Activation Products in Myeloperoxidase- and Proteinase 3-Antineutrophil Cytoplasmic Antibody-Associated Vasculitis. Arthritis Rheumatol..

[99] Ohlsson S., Holm L., Hansson C., Ohlsson S.M., Gunnarsson L., Pettersson A., Skattum L. (2019). Neutrophils from ANCA-associated vasculitis patients show an increased capacity to activate the complement system via the alternative pathway after ANCA stimulation. PLoS ONE.

[100] Brilland B., Garnier A.S., Chevailler A., Jeannin P., Subra J.F., Augusto J.F. (2020). Complement alternative pathway in ANCA-associated vasculitis: Two decades from bench to bedside. Autoimmun. Rev..

[101] Jayne D. (2019). Complement inhibition in ANCA vasculitis. Nephrol. Ther..

[102] Manthey H.D., Woodruff T.M., Taylor S.M., Monk P.N. (2009). Complement component 5a (C5a). Int. J. Biochem. Cell. Biol..

[103] Raby A.C., Holst B., Davies J., Colmont C., Laumonnier Y., Coles B., Shah S., Hall J., Topley N., Kohl J. (2011). TLR activation enhances C5a-induced pro-inflammatory responses by negatively modulating the second C5a receptor, C5L2. Eur. J. Immunol..

[104] Hao J., Huang Y.M., Zhao M.H., Chen M. (2014). The interaction between C5a and sphingosine-1-phosphate in neutrophils for antineutrophil cytoplasmic antibody mediated activation. Arthritis Res. Ther. Ther..

[105] Kallenberg C.G., Heeringa P. (2013). Complement is crucial in the pathogenesis of ANCA-associated vasculitis. Kidney Int..

[106] Chen S.F., Wang F.M., Li Z.Y., Yu F., Chen M., Zhao M.H. (2017). The functional activities of complement factor H are impaired in patients with ANCA-positive vasculitis. Clin. Immunol..

[107] Chen S.F., Wang F.M., Li Z.Y., Yu F., Chen M., Zhao M.H. (2018). Complement Factor H Inhibits Anti-Neutrophil Cytoplasmic Autoantibody-Induced Neutrophil Activation by Interacting with Neutrophils. Front. Immunol..

[108] Cheng L., Gou S.J., Qiu H.Y., Ma L., Fu P. (2018). Complement regulatory proteins in kidneys of patients with anti-neutrophil cytoplasmic antibody (ANCA)-associated vasculitis. Clin. Exp. Immunol..

[109] Choi H., Kim Y., Jung S.M., Song J.J., Park Y.B., Lee S.W. (2019). Low serum complement 3 level is associated with severe ANCA-associated vasculitis at diagnosis. Clin. Exp. Nephrol..

[110] Garcia L., Pena C.E., Maldonado R.A., Costi C., Mamberti M., Martins E., Garcia M.A. (2019). Increased renal damage in hypocomplementemic patients with ANCA-associated vasculitis: Retrospective cohort study. Clin. Rheumatol..

[111] Nguyen Y., Guillevin L. (2018). Eosinophilic Granulomatosis with Polyangiitis (Churg-Strauss). Semin. Respir. Crit. Care Med..

[112] Sinico R.A., Di Toma L., Maggiore U., Bottero P., Radice A., Tosoni C., Grasselli C., Pavone L., Gregorini G., Monti S. (2005). Prevalence and clinical significance of antineutrophil cytoplasmic antibodies in Churg-Strauss syndrome. Arthritis Rheum..

[113] Comarmond C., Cacoub P. (2014). Granulomatosis with polyangiitis (Wegener): Clinical aspects and treatment. Autoimmun. Rev..

[114] Moosig F., Bremer J.P., Hellmich B., Holle J.U., Holl-Ulrich K., Laudien M., Matthis C., Metzler C., Nolle B., Richardt G. (2013). A vasculitis centre based management strategy leads to improved outcome in eosinophilic granulomatosis and polyangiitis (Churg-Strauss, EGPA): Monocentric experiences in 150 patients. Ann. Rheum. Dis..

[115] Valent P., Klion A.D., Horny H.P., Roufosse F., Gotlib J., Weller P.F., Hellmann A., Metzgeroth G., Leiferman K.M., Arock M. (2012). Contemporary consensus proposal on criteria and classification of eosinophilic disorders and related syndromes. J. Allergy Clin. Immunol..

[116] Trivioli G., Terrier B., Vaglio A. (2020). Eosinophilic granulomatosis with polyangiitis: Understanding the disease and its management. Rheumatology.

[117] Kiene M., Csernok E., Muller A., Metzler C., Trabandt A., Gross W.L. (2001). Elevated interleukin-4 and interleukin-13 production by T cell lines from patients with Churg-Strauss syndrome. Arthritis Rheum..

[118] Tsurikisawa N., Saito H., Oshikata C., Tsuburai T., Akiyama K. (2013). Decreases in the numbers of peripheral blood regulatory T cells, and increases in the levels of memory and activated B cells, in patients with active eosinophilic granulomatosis and polyangiitis. J. Clin. Immunol..

[119] Emmi G., Silvestri E., Marconi R., Carrai V., Fanelli T., Zucchini P., Marasca R., Vannucchi A.M., Emmi L., Prisco D. (2015). First report of FIP1L1-PDGFRalpha-positive eosinophilic granulomatosis with polyangiitis. Rheumatolology.

[120] Polzer K., Karonitsch T., Neumann T., Eger G., Haberler C., Soleiman A., Hellmich B., Csernok E., Distler J., Manger B. (2008). Eotaxin-3 is involved in Churg-Strauss syndrome--a serum marker closely correlating with disease activity. Rheumatolology.

[121] Terrier B., Bieche I., Maisonobe T., Laurendeau I., Rosenzwajg M., Kahn J.E., Diemert M.C., Musset L., Vidaud M., Sene D. (2010). Interleukin-25: A cytokine linking eosinophils and adaptive immunity in Churg-Strauss syndrome. Blood.

[122] Jakiela B., Sanak M., Szczeklik W., Sokolowska B., Plutecka H., Mastalerz L., Musial J., Szczeklik A. (2011). Both Th2 and Th17 responses are involved in the pathogenesis of Churg-Strauss syndrome. Clin. Exp. Rheumatol..

[123] Yates M., Watts R. (2017). ANCA-associated vasculitis. Clin. Med. (Lond.).

[124] Rowaiye O.O., Kusztal M., Klinger M. (2015). The kidneys and ANCA-associated vasculitis: From pathogenesis to diagnosis. Clin. Kidney J..

[125] Said M.S. (2010). Upper respiratory tract symptoms, renal involvement and vasculitis: A case report and review of wegener granulomatosis. J. Clin. Med. Res..

[126] Parra-Garcia G.D., Callejas-Rubio J.L., Rios-Fernandez R., Sainz-Quevedo M., Ortego-Centeno N. (2012). Otolaryngologic manifestations of systemic vasculitis. Acta Otorrinolaringol. Esp..

[127] Alba M.A., Flores-Suarez L.F., Henderson A.G., Xiao H., Hu P., Nachman P.H., Falk R.J., Charles Jennette J. (2017). Interstital lung disease in ANCA vasculitis. Autoimmun. Rev..

[128] Watkins A.S., Kempen J.H., Choi D., Liesegang T.L., Pujari S.S., Newcomb C., Nussenblatt R.B., Rosenbaum J.T., Thorne J.E., Foster C.S. (2009). Ocular disease in patients with ANCA-positive vasculitis. J. Ocul. Biol. Dis. Infor..

[129] Demirkesen C. (2017). Approach to cutaneous vasculitides with special emphasis on small vessel vasculitis: Histopathology and direct immunofluorescence. Curr. Opin. Rheumatol..

[130] Wludarczyk A., Szczeklik W. (2016). Neurological manifestations in ANCA-associated vasculitis—Assessment and treatment. Expert Rev. Neurother..

[131] Storesund B., Gran J.T., Koldingsnes W. (1998). Severe intestinal involvement in Wegener’s granulomatosis: Report of two cases and review of the literature. Br. J. Rheumatol..

[132] Berti A., Matteson E.L., Crowson C.S., Specks U., Cornec D. (2018). Risk of Cardiovascular Disease and Venous Thromboembolism among Patients with Incident ANCA-Associated Vasculitis: A 20-Year Population-Based Cohort Study. Mayo Clin. Proc..

[133] Stone J.H. (2003). Wegener’s Granulomatosis Etanercept Trial Research G: Limited versus severe Wegener’s granulomatosis: Baseline data on patients in the Wegener’s granulomatosis etanercept trial. Arthritis Rheum..

[134] Sinico R.A., Bottero P. (2009). Churg-Strauss angiitis. Best Pract. Res. Clin. Rheumatol..

[135] Keogh K.A., Specks U. (2003). Churg-Strauss syndrome: Clinical presentation, antineutrophil cytoplasmic antibodies, and leukotriene receptor antagonists. Am. J. Med..

[136] Mukhtyar C., Lee R., Brown D., Carruthers D., Dasgupta B., Dubey S., Flossmann O., Hall C., Hollywood J., Jayne D. (2009). Modification and validation of the Birmingham Vasculitis Activity Score (version 3). Ann. Rheum. Dis..

[137] Guillevin L., Pagnoux C., Seror R., Mahr A., Mouthon L., Toumelin P.L., French Vasculitis Study G. (2011). The Five-Factor Score revisited: Assessment of prognoses of systemic necrotizing vasculitides based on the French Vasculitis Study Group (FVSG) cohort. Medicine.

[138] Exley A.R., Bacon P.A., Luqmani R.A., Kitas G.D., Gordon C., Savage C.O., Adu D. (1997). Development and initial validation of the Vasculitis Damage Index for the standardized clinical assessment of damage in the systemic vasculitides. Arthritis Rheum..

[139] Chung S.A., Langford C.A., Maz M., Abril A., Gorelik M., Guyatt G., Archer A.M., Conn D.L., Full K.A., Grayson P.C. (2021). 2021 American College of Rheumatology/Vasculitis Foundation Guideline for the Management of Antineutrophil Cytoplasmic Antibody-Associated Vasculitis. Arthritis Rheumatol..

[140] Cupps T.R., Edgar L.C., Fauci A.S. (1982). Suppression of human B lymphocyte function by cyclophosphamide. J. Immunol..

[141] Reinhold-Keller E., Beuge N., Latza U., de Groot K., Rudert H., Nolle B., Heller M., Gross W.L. (2000). An interdisciplinary approach to the care of patients with Wegener’s granulomatosis: Long-term outcome in 155 patients. Arthritis Rheum..

[142] Hoffman G.S., Kerr G.S., Leavitt R.Y., Hallahan C.W., Lebovics R.S., Travis W.D., Rottem M., Fauci A.S. (1992). Wegener granulomatosis: An analysis of 158 patients. Ann. Intern. Med..

[143] Pagnoux C., Mahr A., Hamidou M.A., Boffa J.J., Ruivard M., Ducroix J.P., Kyndt X., Lifermann F., Papo T., Lambert M. (2008). Azathioprine or methotrexate maintenance for ANCA-associated vasculitis. N. Engl. J. Med..

[144] Von Borstel A., Abdulahad W.H., Dekkema G., Rutgers A., Stegeman C.A., Veldman J., Heeringa P., Sanders J.S. (2020). Mycophenolic acid and 6-mercaptopurine both inhibit B-cell proliferation in granulomatosis with polyangiitis patients, whereas only mycophenolic acid inhibits B-cell IL-6 production. PLoS ONE.

[145] Raffray L., Guillevin L. (2020). Rituximab treatment of ANCA-associated vasculitis. Expert Opin. Biol. Ther..

[146] Hofmann K., Clauder A.K., Manz R.A. (2018). Targeting B Cells and Plasma Cells in Autoimmune Diseases. Front. Immunol..

[147] Jones R.B., Tervaert J.W., Hauser T., Luqmani R., Morgan M.D., Peh C.A., Savage C.O., Segelmark M., Tesar V., van Paassen P. (2010). Rituximab versus cyclophosphamide in ANCA-associated renal vasculitis. N. Engl. J. Med..

[148] Unizony S., Villarreal M., Miloslavsky E.M., Lu N., Merkel P.A., Spiera R., Seo P., Langford C.A., Hoffman G.S., Kallenberg C.M. (2016). Clinical outcomes of treatment of anti-neutrophil cytoplasmic antibody (ANCA)-associated vasculitis based on ANCA type. Ann. Rheum. Dis..

[149] Rovin B.H., Caster D.J., Cattran D.C., Gibson K.L., Hogan J.J., Moeller M.J., Roccatello D., Cheung M., Wheeler D.C., Winkelmayer W.C. (2019). Management and treatment of glomerular diseases (part 2): Conclusions from a Kidney Disease: Improving Global Outcomes (KDIGO) Controversies Conference. Kidney Int..

[150] McClure M., Gopaluni S., Jayne D., Jones R. (2018). B cell therapy in ANCA-associated vasculitis: Current and emerging treatment options. Nat. Rev. Rheumatol..

[151] Roberts D.M., Jones R.B., Smith R.M., Alberici F., Kumaratne D.S., Burns S., Jayne D.R. (2015). Rituximab-associated hypogammaglobulinemia: Incidence, predictors and outcomes in patients with multi-system autoimmune disease. J. Autoimmun..

[152] Tieu J., Smith R., Basu N., Brogan P., D’Cruz D., Dhaun N., Flossmann O., Harper L., Jones R.B., Lanyon P.C. (2020). Rituximab for maintenance of remission in ANCA-associated vasculitis: Expert consensus guidelines. Rheumatology.

[153] Zonozi R., Wallace Z.S., Laliberte K., Huizenga N.R., Rosenthal J.M., Rhee E.P., Cortazar F.B., Niles J.L. (2021). Incidence, Clinical Features, and Outcomes of Late-Onset Neutropenia from Rituximab for Autoimmune Disease. Arthritis Rheumatol..

[154] Emadi A., Jones R.J., Brodsky R.A. (2009). Cyclophosphamide and cancer: Golden anniversary. Nat. Rev. Clin. Oncol..

[155] Walsh M., Merkel P.A., Jayne D.R.W. (2020). Plasma Exchange and Glucocorticoids in Severe ANCA-Associated Vasculitis. Reply. N. Engl. J. Med..

[156] Guillevin L., Pagnoux C., Karras A., Khouatra C., Aumaitre O., Cohen P., Maurier F., Decaux O., Ninet J., Gobert P. (2014). Rituximab versus azathioprine for maintenance in ANCA-associated vasculitis. N. Engl. J. Med..

[157] Mohammad A.J., Hot A., Arndt F., Moosig F., Guerry M.J., Amudala N., Smith R., Sivasothy P., Guillevin L., Merkel P.A. (2016). Rituximab for the treatment of eosinophilic granulomatosis with polyangiitis (Churg-Strauss). Ann. Rheum. Dis..

[158] Teixeira V., Mohammad A.J., Jones R.B., Smith R., Jayne D. (2019). Efficacy and safety of rituximab in the treatment of eosinophilic granulomatosis with polyangiitis. RMD Open.

[159] Emmi G., Rossi G.M., Urban M.L., Silvestri E., Prisco D., Goldoni M., Vaglio A. (2018). Scheduled rituximab maintenance reduces relapse rate in eosinophilic granulomatosis with polyangiitis. Ann. Rheum. Dis..

[160] Thiel J., Troilo A., Salzer U., Schleyer T., Halmschlag K., Rizzi M., Frede N., Venhoff A., Voll R.E., Venhoff N. (2017). Rituximab as Induction Therapy in Eosinophilic Granulomatosis with Polyangiitis Refractory to Conventional Immunosuppressive Treatment: A 36-Month Follow-Up Analysis. J. Allergy Clin. Immunol. Pract..

[161] Menditto V.G., Rossetti G., Olivari D., Angeletti A., Rocchi M., Gabrielli A., Pomponio G. (2021). Rituximab for eosinophilic granulomatosis with polyangiitis: A systematic review of observational studies. Rheumatology.

[162] Akiyama M., Kaneko Y., Takeuchi T. (2021). Rituximab for the treatment of eosinophilic granulomatosis with polyangiitis: A systematic literature review. Autoimmun. Rev..

[163] Canzian A., Venhoff N., Urban M.L., Sartorelli S., Ruppert A.M., Groh M., Girszyn N., Taille C., Maurier F., Cottin V. (2021). Use of Biologics to Treat Relapsing and/or Refractory Eosinophilic Granulomatosis with Polyangiitis: Data from a European Collaborative Study. Arthritis Rheumatol..

[164] Terrier B., de Moreuil C., Bonnotte B. (2021). Rituximab versus conventional therapeutic strategy for remission induction in eosinophilic granulomatosis with polyangiitis: A double-blind, randomized, controlled trial. Arthritis Rheum..

[165] Wetsel R.A. (1995). Expression of the complement C5a anaphylatoxin receptor (C5aR) on non-myeloid cells. Immunol. Lett..

[166] Bekker P., Dairaghi D., Seitz L., Leleti M., Wang Y., Ertl L., Baumgart T., Shugarts S., Lohr L., Dang T. (2016). Characterization of Pharmacologic and Pharmacokinetic Properties of CCX168, a Potent and Selective Orally Administered Complement 5a Receptor Inhibitor, Based on Preclinical Evaluation and Randomized Phase 1 Clinical Study. PLoS ONE.

[167] Jayne D.R.W., Bruchfeld A.N., Harper L., Schaier M., Venning M.C., Hamilton P., Burst V., Grundmann F., Jadoul M., Szombati I. (2017). Randomized Trial of C5a Receptor Inhibitor Avacopan in ANCA-Associated Vasculitis. J. Am. Soc. Nephrol..

[168] Merkel P.A., Niles J., Jimenez R., Spiera R.F., Rovin B.H., Bomback A., Pagnoux C., Potarca A., Schall T.J., Bekker P. (2020). Adjunctive Treatment with Avacopan, an Oral C5a Receptor Inhibitor, in Patients with Antineutrophil Cytoplasmic Antibody-Associated Vasculitis. ACR Open Rheumatol..

[169] Jayne D.R.W., Merkel P.A., Bekker P. (2021). Avacopan for the Treatment of ANCA-Associated Vasculitis. Reply. N. Engl. J. Med..

[170] Kitamura F., Yamaguchi M., Nishimura M., Katsuno T., Ito M., Sugiyama H., Iwagaitsu S., Nobata H., Kinashi H., Ishimoto T. (2022). Anti-neutrophil cytoplasmic antibody-associated vasculitis complicated by thrombotic microangiopathy with posterior reversible encephalopathy syndrome successfully treated with eculizumab: A case report. Mod. Rheumatol. Case Rep..

[171] Manenti L., Urban M.L., Maritati F., Galetti M., Vaglio A. (2017). Complement blockade in ANCA-associated vasculitis: An index case, current concepts and future perspectives. Intern. Emerg. Med..

[172] Ribes D., Belliere J., Piedrafita A., Faguer S. (2019). Glucocorticoid-free induction regimen in severe ANCA-associated vasculitis using a combination of rituximab and eculizumab. Rheumatololgy.

[173] Riedl M.A., Grivcheva-Panovska V., Moldovan D., Baker J., Yang W.H., Giannetti B.M., Reshef A., Andrejevic S., Lockey R.F., Hakl R. (2017). Recombinant human C1 esterase inhibitor for prophylaxis of hereditary angio-oedema: A phase 2, multicentre, randomised, double-blind, placebo-controlled crossover trial. Lancet.

[174] Molfino N.A., Gossage D., Kolbeck R., Parker J.M., Geba G.P. (2012). Molecular and clinical rationale for therapeutic targeting of interleukin-5 and its receptor. Clin. Exp. Allergy.

[175] Wechsler M.E., Akuthota P., Jayne D., Khoury P., Klion A., Langford C.A., Merkel P.A., Moosig F., Specks U., Cid M.C. (2017). Mepolizumab or Placebo for Eosinophilic Granulomatosis with Polyangiitis. N. Engl. J. Med..

[176] Tsurikisawa N., Oshikata C., Watanabe M., Fukuda N., Yamaguchi T., Kiyohara H., Kaneko T. (2021). Clinical Features of Patients with Active Eosinophilic Granulomatosis with Polyangiitis Successfully Treated with Mepolizumab. Int. Arch. Allergy Immunol..

[177] Rios-Garces R., Prieto-Gonzalez S., Hernandez-Rodriguez J., Arismendi E., Alobid I., Penatti A.E., Cid M.C., Espigol-Frigole G. (2022). Response to mepolizumab according to disease manifestations in patients with eosinophilic granulomatosis with polyangiitis. Eur. J. Intern. Med..

[178] Caminati M., Crisafulli E., Lunardi C., Micheletto C., Festi G., Maule M., Giollo A., Orsolini G., Senna G. (2021). Mepolizumab 100 mg in severe asthmatic patients with EGPA in remission phase. J. Allergy Clin. Immunol. Pract..

[179] Manka L.A., Guntur V.P., Denson J.L., Dunn R.M., Dollin Y.T., Strand M.J., Wechsler M.E. (2021). Efficacy and safety of reslizumab in the treatment of eosinophilic granulomatosis with polyangiitis. Ann. Allergy Asthma Immunol..

[180] Guntur V.P., Manka L.A., Denson J.L., Dunn R.M., Dollin Y.T., Gill M., Kolakowski C., Strand M.J., Wechsler M.E. (2021). Benralizumab as a Steroid-Sparing Treatment Option in Eosinophilic Granulomatosis with Polyangiitis. J. Allergy Clin. Immunol. Pract..

